# A Direct Reporting Platform for Financial Exploitation: From Banks to APS

**DOI:** 10.1093/geroni/igaa057.2438

**Published:** 2020-12-16

**Authors:** Jason Burnett, Sally Reisch, Geoffrey Rogers

**Affiliations:** 1 McGovern Medical School at UTHealth, Houston, Texas, United States; 2 Hunter College, New York, New York, United States

## Abstract

Despite encouraging changes in regulations and practices, opportunities remain to strengthen the collaboration between Financial Services Institutions (FSI) and Financial Exploitation (FE) investigative agencies such as Adult Protective Services (APS). A major barrier to these collaborations is timely and effective information exchange, between the agencies, for maximizing client protection. Often times, the need for more information from the agencies involved delays or mitigates the provision of financial protection to the client/victim. Through the U.S. Office for Victims of Crime, funding has been provided to develop a single reporting platform for enhancing communication and collaboration between FSI and APS. The helpful platform provides an innovative conduit for providing timely and effective information exchange between FSI and APS agencies to better serve and protect older adults against FE. This presentation will discuss the platform development, the lessons learned, preliminary data and future research. Part of a symposium sponsored by Abuse, Neglect and Exploitation of Elderly People Interest Group.

